# Exploring young women’s reproductive decision-making, agency and social norms in South African informal settlements

**DOI:** 10.1371/journal.pone.0231181

**Published:** 2020-04-29

**Authors:** Samantha Willan, Andrew Gibbs, Inge Petersen, Rachel Jewkes

**Affiliations:** 1 Gender and Health Research Unit, South African Medical Research Council, Pretoria, South Africa; 2 Centre for Rural Health, School of Nursing and Public Health, University of KwaZulu-Natal, Durban, South Africa; Queen’s University at Kingston, CANADA

## Abstract

This paper explores reproductive decision-making among young women in South Africa’s informal settlements and considers whether and how agency and social norm theory inform their decisions. Understanding whether, when and how young women make decisions about conception and motherhood is critical for supporting women to avoid unplanned, early motherhood. Qualitative data were collected from 15 young women in informal settlements in eThekwini, South Africa at three time points over 18 months, using in-depth interviews, participant observation and photovoice, and were analysed inductively. When the young women were teenagers and into their early twenties, and had not yet had a child, most paid little attention to whether or not they conceived. This shifted as they grew older and/or after having a first child, at which point many of the women began to express, and sometimes act upon, a greater desire to control whether and when they conceived and delay further pregnancies. At different times in their lives, both social norms and reproductive agency, specifically ‘distributed agency’ played significant roles in influencing their reproductive decision-making. Social norms held the most influence when they were teenagers and experiencing normative pressures to have a baby while young. As they grew older and/or had a first child they began to assert some agentic control around their reproduction. We therefore recommend that in order to improve the effectiveness of services and interventions supporting young women to delay unplanned pregnancies, programmers, researchers and policy makers must develop a better understanding of the role of social norms and agency at different stages of women’s lives.

## Introduction

Many young women in South Africa do not make decisions about whether, when and how many children to have; which we refer to as limited reproductive decision-making. Reproductive decision-making involves decisions about parenthood (whether and when to be a parent, and the number and spacing of children one wishes to have), including decisions around contraceptive usage and fertility [1]. While adolescent motherhood has declined since the 1980s [2, 3], a third of young South African women still experience pregnancy before the age of 20 [4], and ‘young families’ including young children, young mothers and young grandmothers remain common [5]. At the same time accurate and consistent contraceptive use remains low, Chersich et al (2017) found only 49% of South African women (15–49 years) who had ever had sex used contraceptives, with lower use among black Africans and younger populations [6]. In order to develop appropriate services and interventions to strengthen young women’s reproductive decision-making it is important to understand whether, when and how young women make reproductive decisions, and how reproductive agency and social norms influence these decisions.

Much literature on reproductive decision-making focuses on strengthening women’s individual agency as a way to support them to use contraceptives effectively and to be in control of their fertility and whether and when they have children [7, 8]. Kabeer (1999) defines agency as one’s ability to identify one’s own goals and act upon them [9]. In the context of reproductive decision-making, reproductive agency means being able to set individual reproductive goals and follow through with actions to realise the goals. This would include reproductive goals about whether, when and how many children to have, and being in a position to effectively use contraceptives and pregnancy terminations to control fertility, to enable women to realise their goals. Thus agency assumes that people not only set goals, but also take action to achieve these goals, referred to as ‘agentic actions’.

However, there is a debate regarding what constitutes ‘agentic actions’. Mahmood (2001) and Madhok et al (2013) suggest that the over-focus on clear, measurable ‘agentic actions’ ignores the strategies and actions that women take in challenging contexts [10, 11]. Building on this Campbell and Mannell (2016) [12] used this work to reflect on women’s agency in relation to experiences of intimate partner violence (IPV). They questioned the widely held view that in order to have agency women must always take bold steps, which in the case of IPV is often seen to mean leaving or reporting a violent partner. Campbell and Mannell (2016), argue instead for what they call ‘distributed agency’ which recognises more nuance and recognition of “‘small wins’ that are realistically achievable by real women in real situations” (p. 22). Furthermore, distributed agency argues against the idea that a woman either ‘has agency’ or ‘does not’, rather it recognises that agentic decisions are fluid and contingent on multiple factors, in some moments a woman may have agency over certain decisions and in another moment, or a different context, the same woman may not.

Many researchers argue that strengthening reproductive agency is important for women as it enables them to make and act upon decisions including conception, early motherhood and modern contraceptive use, resulting in lower fertility, longer birth intervals and fewer unplanned pregnancies [7, 8, 13]. A growing number of authors argue similarly to Campbell and Mannell (2016), that women who make reproductive decisions often do not take bold steps, rather choosing small, yet meaningful steps to delay pregnancy, frequently through secretly using contraceptives or avoiding sex. Furthermore, they argue that even some women in violent relationships succeed in making reproductive decisions [14–16]. Mkandawire-Valhmu et al (2016) writing about women in Malawi highlight this arguing strongly that the secret use of contraceptives is an important act of resistance, enabling women to effectively assert their reproductive agency often within violent, patriarchal relationships.

Nonetheless, many women have limited reproductive agency, particularly where they experience intimate partner violence (IPV) and/or have controlling partners. Constrained reproductive agency often leads to: reproductive coercion (forced sex to conceive, contraceptive control and pregnancy pressure), constrained contraceptive use, and repeat unplanned pregnancies [7, 16–19]. Globally, reproductive coercion is widespread among adolescents and adults, ranging from 15–25%, with higher prevalence among women exposed to IPV [20].

Alongside research on reproductive agency is a body of literature exploring the impact of social norms, highlighting the influence of groups and normative behavior on individual decision-making [21–23]. Young (2015) asserts that social norms act as informal rules influencing how we behave, informing what we believe others expect us to do and our expectations about what others will do. Cislaghi & Heise (2018) note that social norms significantly influence peoples’ choices and behaviors on health-related issues, and people conform to norms because of anticipated “rewards or punishments for compliance and non-compliance respectively” (Cislaghi & Heise, 2018, p. 2). The Theory of Planned Behavior (TPB) also offers insight into what informs individual’s behaviors, arguing that three factors are important: the individual’s attitude towards a behavior, subjective norms about the behavior and the individual’s perceived control over the behavior (agency) [24]. Fishbein (discussed in Montano & Kasprzyk, 2008) highlighted that individual’s underlying beliefs often differ for similar actions, such as holding different beliefs about using a condom with a casual versus a main partner, similarly he notes that they differ for different populations and settings.

The prevalent social norms influencing reproductive decision-making in South Africa encourage young motherhood in order to prove fertility, creating encouragement and/or pressure on young women to comply. This pressure comes from families, friends, community, church and partners [3, 16, 25–28]. Yet there is also a competing narrative against young motherhood, maintaining that it is a sign of recent moral decay, however this is not the dominant normative view in most communities [27]. Furthermore, Mkhwanazi and Bhana (2017) noted that young women’s voices are seldom heard in this narrative, calling for more work to highlight young women’s perspectives.

These competing pressures are also very evident around adolescent sexuality. While adolescent sexuality is partly taboo and condemned, at the same time early sex is often tolerated and early motherhood, especially in KwaZulu-Natal, South Africa, is widely accepted, despite initial disappointment and anger in some situations, and does not carry severe sanction [5, 26, 29]. Many traditional practices and ceremonies for adolescent girls in KwaZulu-Natal also provide ambiguous messages, differing across communities some are designed to inform young girls about their sexuality and expected behaviors, such as the “*ukumisa iduku*” ceremony, a practice led by the community elders that recognises that young people have relationships and which provides them with knowledge about sex. While others are designed to inhibit adolescent sexuality, such as “*virginity testing”*, where the elders check young women’s hymens to pronounce whether they are still virgins. Virginity testing is still common in many rural settings, but is seldom used in urban settings, highlighting how these traditions and normative expectations shift over time and space, indeed many young women may never participate in these ceremonies, while others may experience both [14].

Previous authors have argued that young women’s experiences of reproductive decision-making often shifts after having a first child, whereby after a first child a women can make more decisions around timing and spacing of future children [4]. At this point many women express desires to delay future pregnancies until they are in a better position to care for their children, either through marriage, completing schooling or securing a job [4]. Wood and Jewkes (2006) and Willan (2013) found that many young women are only provided with comprehensive and accurate contraceptive knowledge and options after a first birth, for many this is the first time they are supported by healthcare providers to prevent and/or plan conception. However, Preston-Whyte and Zondi (1989) note that this reproductive decision-making is usually lost once again following marriage, when normative pressure for more children is re-asserted, in many cases if a new wife does not conceive quickly she is questioned and often ridiculed. At this point the pressure to conceive generally comes from the family to ensure lineage and if the husband is not the father of her existing child(ren), it is also to prove his virility.

The literature on women’s agency identifies two key approaches to strengthening their decision-making. The first, supporting women to build their individual agency by enabling her to set goals and act upon them [9]. The second considers that women’s individual choices and actions (her agency) is significantly influenced and constrained by the dominant social norms [19, 21–23]. While the agency literature specifically reflects on strengthening reproductive decision-making, social norms has only recently been considered in the reproductive-decision making literature, there is also very little that looks at how the two intersect. Understanding the role of both approaches and how they intersect is critical for informing the appropriate development of interventions and services to support young women to make agentic reproductive decisions at all stages of life. The aims of this study was thus to explore which women exercise reproductive decision-making and choose to avoid early motherhood, why they do this and what enables them to do so, and conversely to understand the experience of women who do conceive at a young age.

This paper seeks to explore young women’s reproductive decision-making through interviews with fifteen young women living in informal settlements in eThekwini, South Africa. The data shows that at different times in their lives both social norms and agency played significant roles in influencing women’s reproductive decision-making. Many of the young women did not make agentic decisions about their first child and becoming young mothers, due mostly to normative pressure to conceive young to prove fertility. However, for some young women this shifted when it came to their second child and they grew older, at which point more of the young women, although not all, were able to assert reproductive agency and delay further pregnancies. The normative pressures to conceive, although not completely negated, had less influence at this point and individual choices and agency became more pronounced. Furthermore, for many young women controlling their bodies and reproductive decision-making was not a key concern for them, raising questions about the relevance of the agency and social norms theories for these young women.

## Methods

### Methods and setting

Multiple-case study research was conducted with fifteen young women participating in a randomised control trial (RCT) in two informal settlements in eThekwini Municipality, South Africa. The trial aimed to reduce IPV and HIV-risk through implementing the Stepping Stones and Creating Futures intervention. The RCT had thirty-four clusters randomized into control and intervention, it enrolled 1360 women and men and collected quantitative data at baseline, 12 and 24 months. Qualitative data was collected with men (19 at baseline, 5 were followed up at 12 and 24 months) and women, this paper focuses on the latter. [30]. Qualitative data were collected from the fifteen women at three time points, baseline (May 2016 –February 2017), 12 months and 18 months follow up. Each women’s experience over time formed one case study. Semi-structured, in-depth interviews were undertaken, supplemented by photovoice and ongoing participatory observations. These qualitative methods were chosen for their value in meaningfully including marginalised voices, and supporting an in-depth understanding of the women’s perspectives and lives [31]. The study design and analysis meets the Consolidated Criteria for Reporting Qualitative Research (COREQ) guidelines [32].

Participants were drawn from two intervention communities, purposively selected for ease of access, safety and feasibility of undertaking the research [30]. The women were recruited through convenience sampling, and eligibility for inclusion included: not being formally employed, aged between 18–30 years and not in school. The sample size was determined through purposeful sampling. All women from the intervention groups in the two selected communities were invited to join the qualitative research, and all agreed to participate.

The first two authors and last author were involved in research design and data analysis, they are experienced researchers in the field with the second, third and fourth authors holding Doctorates, the first author is currently completing her Doctorate in Public Health. The third author has extensive experience in challenging contexts and mental health issues. Data collection was undertaken by one Research Assistant who was completing her MSc and had training in data collection and research methods. She was carefully selected for her skills, experience and compatibility to the participants; she was a similar age to the participants, an isiZulu speaker from eThekwini with a good socio-political understanding of the context. She was closely monitored by the first two authors, which included regular de-briefings, reviewing of transcripts and trouble-shooting. All interviews, participant observations and photovoice workshops were undertaken by the Research Assistant, the latter supported by the first author.

The two informal settlements were very similar, enabling comparability across themes. Residents in both had low education levels, were very poor with high rates of unemployment, experienced over-crowding, and there was limited or no formal housing or services. Despite similarities there were a few differences in relation to housing and access to water. One had slightly better housing and taps in the yards, while the second had more dilapidated housing and shacks, and only communal taps.

### Data collection

All women were interviewed twice at baseline, at 12 months twelve women were interviewed, and at endline thirteen were interviewed, a few women were lost to follow up as they moved far away. The first baseline interview involved open-ended questions about the participants’ lives and focused on building trust. The second, usually conducted within two weeks of the first interview, focused on in-depth discussions about relationships, conception, pregnancy, contraceptive use, motherhood, abortion, experiences of violence and decision-making. Each interview took between 45 and 90 minutes. Midline and endline interviews were of similar length and focused on the same topics, reflecting on any changes and how and why such changes occurred. See S1 and S2 Appendices for interview guides.

Participant observation was undertaken with the seven women from the one community throughout the 18 month research period. The Research Assistant spent approximately 80 hours in the community talking and observing the women’s daily experiences. Relevant observations were written up, in English, and became a critical third source of data, supplementing the IDI’s and photovoice process. These observations enhanced the research teams understanding of the specific community dynamics and the women’s lives.

Eight women in the other community, participated in photovoice. This participatory technique involves women taking photographs, followed by facilitated group discussions about the photographs to explore and reflect on women’s lived experiences, and to imagine futures [33]. At baseline three, three-hour workshops were held, with two periods in between for taking photos. At endline two, four-hour workshops were held with one period for taking photos. The sessions were facilitated in isiZulu by the Research Assistant, with support from the first author. Sessions where participatory with the women encouraged to discuss specific topics to stimulate reflections and ideas of what to capture with photographs. They were then provided with inexpensive cameras to take photos for the next session, they were given training on how to use them. In subsequent sessions the women selected their photos which were immediately printed and they created photo-posters which they presented and discussed with the group. Focus areas for the discussions and photos included: power, goals and dreams, relationships, pregnancy, and motherhood, during the final sessions they were asked to reflect generally on their lives, in particular any changes and pathways to change. The women produced 28 photo-posters of their lives. See S3 Appendix for the photovoice guide. Interviews and the photovoice workshops were conducted in isiZulu, by the female Research Assistant, then translated and transcribed into English.

### Data analysis

Data were analysed inductively, which allowed themes and sub-themes to gradually emerge from the data itself through close reading of the transcripts 2010 [34, 35]. The different data sources were initially analysed separately, then triangulated vertically (baseline, midline and endline) for each individual case. Thereafter they were analysed horizontally across all cases to extract common themes across the participants. All data was analysed at 18-months. Inductive analysis enabled the researchers to identify the women’s experiences, beliefs, and goals related to reproduction and agency in their lives. Inductive coding lead to initial themes being created on conception and babies, motherhood, contraceptive knowledge and use, relationships and IPV. A second level of coding then generated sub-themes and connections between themes, these wove together to form the narrative for the paper, the sub themes were selected for frequency, and importance that the women placed on them.

The researchers took care to ensure rigorous data collection and analysis. Recognising our positionality, including the inevitable power imbalances between interviewer and interviewee, self-reflexivity was used to avoid and correct for this, as much as possible. as Wilkie notes through regularly reflecting on ourselves as researchers and the research process itself, we were able to “reduce the risk of being misled by our own experiences and interpretations.” [36]

To do this we used a few strategies, first, open inductive approaches were used to generate ground-up understandings, rather than impose a top-down structure on the data. Second, the team spent over two years in the field, developing an extensive contextual understanding of the communities. Third, interview guides were carefully structured to avoid social desirability and enable participant reflections and avoided leading responses, we also encouraged discussions about close friend’s behaviors which sometimes facilitates more candid discussions around sensitive topics. Forth, we used triangulation of IDI, photovoice and participant observations, which allowed similarities and differences to emerge. Fifth, we selected the research team very carefully, the Research Assistant who had primary contact with the participants, was of similar age, spoke the same language and was familiar with the area, although we acknowledge that this did not completely negate her position of power due to her higher socio-economic status. The first author, who is significantly older, and not a first language isiZulu speaker did not directly engage with the community nor the young women, except during the photovoice sessions, where she took a backseat supporting the Research Assistant when requested. Finally, the analysis was undertaken from an explicitly feminist viewpoint, the team was committed to a meaningful analysis that revealed the women’s true stories as experienced and told by the women themselves.

### Ethics

Ethical approval was received from the Biomedical Research Ethics Committee (BREC) at the University of KwaZulu-Natal, Durban, South Africa (BFC043/15) and the South African Medical Research Council Ethics Committee (EC006-2/2015). All women signed informed consent forms for IDIs and photovoice sessions. The trial was registered on 13 January 2017, number NCT03022370.

All names used are pseudonyms.

## Findings

Demographic data from the larger study indicates that the informal settlements where the young women lived were characterised by extreme poverty, gender inequality, limited employment prospects and generalised violence [37]. The women were aged between 19–29 years at baseline and IPV among them was common, twelve of the fifteen women experienced emotional, physical, economic and/or sexual violence within their relationships, and controlling behaviors from male partners were widespread. Nearly all of the women were in established intimate heterosexual relationships, some had an occasional casual partner(s) in addition to their main partner, only one women was married, most women lived with family members, occasionally living with partners. None were in formal employment and only two had completed secondary school.

Eight of the young women were mothers at baseline, three of whom had more than one child. During the research period two women became pregnant and both had their first babies between the 12 and 18 month interviews. Five women had conceived but miscarried, and only two of the young women had never been pregnant. No termination of pregnancies were reported by any of the participants. The youngest age of first conception (including miscarriages) was 15, another two participants conceived under the age of 18, in three cases the exact age of conception was unclear.

The data were examined to understand whether, when and how the young women made decisions about whether or not to have babies. It showed that nearly all of the women who conceived young (teenagers through to early twenties), conceived due to socially normative pressures: they did not plan to conceive and did not make reproductive decisions.

### Conception was usually unplanned

The majority of women, including those without children, described at times having limited control over whether or not they fell pregnant, particularly with their first pregnancy and when they were younger (teenagers through to early twenties). The women spoke repeatedly about pregnancy and motherhood being unplanned, mentioning that it “just happened”. Furthermore, they were often ambivalent about conceiving and when they did conceive, they tended to accept it. Olwethu (aged 26, one child), who conceived for the first time when she was 20 told us this:

It’s not a decision [falling pregnant]. I understand it as something that happens when the time comes. Because there are people who have sex but they can’t have children, even though they want them. You can’t control when or when not to have a child, it happens when the time comes.

Sthelo (aged 26, no child) similarly supported this view this: “Having a child will happen organically, I won’t say to him [my partner], ‘hey let’s have a child’. No. It will happen on its own.”

Many of the women also held strong beliefs that the ancestors or God decided on conception. Zoleka (aged 23, no child) illustrated this when she said: “my ancestors are my contraceptives…. My ancestors don’t want me to have a child. I will have a child when they decide that I am ready to have one.”

There were, however, a few women who described having some control, or desire to make decisions about conceiving, and in almost all cases where the women stated they did not wish to conceive they already had a child and/or they were a little older. However, even among this group some were unable to prevent conception while others remained ambivalent about whether they really wanted to prevent future pregnancies. Some women clearly said they did not want to conceive, but then contradicted themselves by saying that if they did conceive they would accept, and even embrace, it. For instance, Zoleka (aged 23, no child) was inconsistent throughout her in-depth interviews and discussions in photovoice sessions, she moved back and forth between wanting and not wanting a baby. She expressed very strong views at stages over the 18 month research period about not wanting children, however she believed her ancestors would determine if she conceived, and she used condoms only occasionally with no other contraceptives. Additionally, she often made remarks such as “if I were to have a child now, it would not be a mistake”. Her inconsistency appeared to reflect both her constantly changing view on motherhood as well as a sense that she was not really able to control her fertility.

Despite some women not being able to prevent unwanted pregnancies, a few women successfully prevented them. Noluvuyo (aged 19, one child) who had her first child at 17 years, expressed very strong views that she would delay further children, her partner who was violent towards her wanted more children, however she took contraceptives secretly, albeit inconsistently. Despite not wanting to conceive at this stage, Noluvuyo added that if she did conceive she would accept it, although this acceptance was discussed with a sense of resignation rather than joy. Thobile (aged 21, no child) spoke with pride about having successfully avoided conceiving, saying she would only have a baby once married or financially established “once I have achieved everything and I know that I have everything I need … I am actually avoiding having a baby without having money.” However, despite very clear and consistent view about not wanting to conceive she used no hormonal contraceptives and only used condom inconsistently, despite being sexually active for a number of years. During the research period she made the first clear decision that would support her to avoid conceiving, she relocated to stay with extended family nearly 100 kms away from her friends and previous partners. She believed that the new environment would support her to continue her schooling and construct a different life for herself. She credited her views to her family’s very strong moral and religious views on ‘proper behaviors’, the normative expectation among her family and immediate community was to avoid early motherhood.

Only one woman, Sebenzile (aged 28, one child) had consciously chosen to conceive. Her partner had wanted a baby for a long time however she had not felt ready to be a mother and had secretly used the hormonal injection to prevent pregnancy. However, at the age of 24, she felt she was ready to have a baby and was happy to become a mother, and to conceive with her partner. When asked if she wanted to be pregnant she replied “Yes I had wanted to have one [a baby].”

Among women who had children, we saw a slightly different focus and conversation emerge, many now talked about choosing the spacing and timing of future children, and delaying future pregnancies. They began to plan whether and when they would have more children, and tried to find ways to act on these plans, although not always successfully, as taking steps to realise these plans remained challenging for many. Some women expressed sadness and regret about not being able to care and provide for their first baby, in at least four cases the babies lived with an aunty or grandmother which made the women sad as they wanted to care for their children. As a result many said they would delay further children until they could provide and care for them. This included the hope of finishing secondary school or occasionally tertiary studies, and finding a good job to enable them to be the primary carer. Enhle, who was 18 when she had her first child, and had returned to school one month after childbirth, expressed this when asked whether she wanted a second child:

Not now. I will think about it [having another child] when I am working. When I am able to support and look after my children. I don’t want my child to live the same life as me. She [my child] needs a better life, I want her to finish school.

To prevent conception Enhle initially used the hormonal implant, however she did not enjoy using it and tried unsuccessfully to have it removed. The clinic staff said they could not remove it as they were not trained to do so, and that she must see for the doctor, after returning numerous times she eventually gave up; “The implant expired. I went to the clinic to have it removed, they [clinic staff] kept turning me back, saying Mr Xulu [the doctor] is not available, he is sick and so forth. Then I gave up.” Despite still having it she began to also use the hormonal injection, and condoms inconsistently.

Sebenzile, who planned her first child, noted that motherhood was hard and said she would not have a second child now “…having a [second] baby is not an option at the moment. It’s better to have a baby when you have a stable job and you know you can hire a nanny and work freely.” Nonetheless, she used contraceptives inconsistently, and remained fatalistic about future conception going on to say: “Anyway pregnancies happen all the time. It depends on your womb, if you are fertile or your womb is weak you will be pregnant no matter what you do.” Others mentioned waiting until *lobola* (bride price paid by the man and his family) was paid, or marriage, at which point they expected the father to support the child, and then they could have more children knowing he would provide for them.

Apart from the few women who talked about delaying pregnancies, for the majority of the women whether or not to have a baby was not something they thought about or talked about, it was not a key concern for them. When the women reflected on what was important in their lives there was very little discussion about consciously planning whether, when and how many babies to have. Discussions during participant observations and photovoice sessions reinforced this, again showing that the women were significantly more interested in talking about their partners, who was dating who, IPV and having fun, or how to find work and make money. Planning, or avoiding, motherhood did not arise as an important issue for most of these young women.

### Inconsistent and inaccurate contraceptive knowledge and use

Most of the young women did not use contraceptives consistently and effectively. In many instances the barriers to effective contraceptive use were around knowledge (limited or misinformation and myths), fear of being mistreated by healthcare providers, and a lack of appropriate options and poor access. In some cases the women used contraceptives ineffectively, despite having knowledge and access, as they were ambivalent around conception, on the one hand wishing to prevent conception while also accepting that despite their wishes they may conceive. After young women had a baby there was a slight shift with some using contraceptives more, although for many this remained inconsistent.

Three key reasons for ineffective contraceptive use were shared. First, a number of young women reported insufficient knowledge about fertility and effective contraceptive use. This was observed during the intervention session on condom use, where many participants commented that they did not know how to put a condom on. Many women commented on how informative the intervention sessions on reproduction and fertility had been, highlighting their limited knowledge prior to the sessions. When Ndoni (aged 25, two children) was asked which session she enjoyed the most she mentioned the session that helped her to understand her own menstrual cycle and fertility and how to use condoms. “[she enjoyed hearing about] what happens when a woman has her period and how she gets pregnant. And how to be careful when using condoms like checking for the expiry date.” Two women who had tubal ligations, did not understand the procedure and continued to use hormonal contraceptives (inconsistently) to prevent conception. Furthermore, local beliefs around fertility and preventing conception often provided young women with inaccurate information, including that one cannot conceive when breastfeeding and that for a number of reasons one cannot use contraceptives until after having a first child. Thobile, who does not have a child, said she did not use contraceptives as it would age her body “I don’t want to prevent [conception] when I don’t have children yet because it’s been said that if you do so without children you will age… They say your body becomes saggy.”

Second, women discussed the challenges of effectively and consistently using contraceptives. They mentioned that they took the hormonal pill irregularly, missed return dates for the hormonal injection and sometimes stopped using contraceptives because of negative side-effects including menstrual irregularities. Many women found it was “just too hard” to use hormonal contraceptives effectively. Olwethu (26 years, one child) said she did not want more children, yet she stopped using hormonal contraceptives relying on inconsistent condom use, she had tried the implant, hormonal pill and injection, and found that none suited her.

The last time we spoke you told me that you removed the hormonal implant and secretly started taking the [hormonal] pill and you hid this information from your boyfriend, what has happened since then, are you still using contraceptives?

I don’t use anything…I don’t use the injection, I stopped the [hormonal] pill. I used the injection for a while and stopped. I am tired [of using contraceptives] and no longer using anything.

**Why did you stop it?**

I was tired of going to the clinic every time [for the renewal] and I stopped, we are strongly using the protection (condoms) [However, she later said this is very inconsistent]

Nkanyezi (mid 20’s, 3 children) started using the hormonal injection after her first child was born, but stopped because of excessive bleeding. Despite not wanting to have more children she did not use any other contraceptives and conceived twice more, thereafter she elected to have a tubal ligation.

Third, young women reported that the clinics were inaccessible (far away, with limited opening hours), often did not have contraceptives in stock and the healthcare workers were frequently judgmental when young women asked for contraceptives. When Ntombi (aged 21, 1 child) became sexually active at 15, her sister and mother did not want her to conceive, and her sister took her to the clinic to access contraceptives, however it was closed, she was frustrated and never returned, and she conceived shortly thereafter. Many women also mentioned how the judgmental healthcare workers discouraged them from asking for contraceptives as they feared being humiliated for being sexually active.

After a first child most women reported increased contraceptive use, which was consistent with the women’s discussions where after having a child they began to be more conscious about planning whether and when to have more children. Despite this increased desire to delay a second pregnancy, and a rise in contraceptive use, nearly all women continued to report inconsistent or inaccurate contraceptive use, leading to many not being able to consistently prevent future pregnancies. Most of the barriers to effectively preventing conception noted above still applied for these young mothers, notwithstanding their intentions to prevent pregnancies. Despite most of the women having much clearer plans to prevent future pregnancies at this point, the barriers and ambivalence still remained for some.

Young women who were mothers had very different experiences when engaging with healthcare workers around contraceptives. Healthcare workers almost always treated young women differently once they were mothers, they now played a significant role in starting women on hormonal contraceptives, although often without providing sufficient information for ongoing effective use. After delivery, healthcare workers almost always put women on the contraceptive injection, on occasions without full and informed consent. However, many women failed to return regularly for follow-up injections. Two women had tubal ligations after childbirth to avoid further pregnancies.

### Social norms drive early motherhood

When reflecting on what was driving early motherhood in these communities, the women described a dominant social pressure within their communities that young women should have a child to prove her fertility, with some discussing a fear that no-one would marry them if they did not have a baby. Many women noted that in order to be considered eligible for marriage it was necessary to have proven their fertility (through having a baby). In addition, if a woman got married, and did not have a baby with her husband, there was also pressure to have a child with him. They also mentioned their desire to conform and ‘fit- in’ with peers who had babies. Indeed, nine of the young women reported that they had felt pressured by family, friends, community and the church to become mothers young.

Nkanyezi (mid 20’s, three children) highlighted how the pressure operated from many sources including the young women themselves. She noted the abuse young women experienced from family, friends and the broader community if they were childless, often being referred to as barren. She also highlighted the women’s own anxieties that she may be barren, and that this would mean she could never conform to the expectations of being a mother. She also noted how her friends spoke admiringly about the women who did have children:

What makes women have children?

Maybe you see your friends having two, three children and you say why not me?

Oh so you see others?

Yes.

Tell me a story of someone you know, a woman here in the community or your friend who took a decision to have a baby because she saw her friends?

Maybe in the community she sees that I’m the only person who does not have a child while others have four, it means I am barren.

What do they say about a woman who can’t have children?

They say you can’t have children, they call you [unkind] names.

The one who has many children, what do they say about her, maybe she is not married she has children out of wedlock?

She is producing [children].

The pressure from friends to conform and have a child came up repeatedly. During a photovoice session Ndoni (aged 25, two children) pressured Zoleka (aged 23, no children) to have a child: “I am saying we are age-mates, by now you should have had a child…” In some cases the women without babies saw their friends enjoying their babies and motherhood, and said they wanted to experience that joy too. When Nkanyezi (mid 20’s, three children) was asked if she had wanted her first child she replied:

At that time we played dolls (laughs) I got a talking doll [a baby] and I was happy.

And the second child?

I wanted it [the child] because I saw people having three children and I though why not, why should I only have one child.

The normalisation of adolescent motherhood was evident in the way families responded to adolescent pregnancies and the babies. In all cases the maternal family, and sometimes the paternal family, came to terms with the adolescent pregnancy and the baby was welcomed, although sometimes this was after initial disappointment from parents. In a few of the cases if parents had the means, they provided support to the young mother and her baby. Noluvuyo, who was 17 when she gave birth, returned to school when the baby was young, and despite having a difficult relationship with her family they cared for her baby to enable this.

However, following a first child, the pressures on women to have more children shifted, they were not expected to have more children before becoming financially stable, married or having *lobola* paid. After proving her fertility, a woman was expected to be responsible, and make clear choices and plans around further children, including considering who will look after her children. As Ntombi (aged 21, one child) illustrated, leaving children with family was often fraught, and was considered irresponsible once it was more than one child:

You can have one child, but two that is an extreme mistake…. No, the first child is okay, that is expected of you as woman … More than one is too much, where are all these children going to sleep…No I think that’s being irresponsible.

Once married or *lobola* was paid, the normative expectations around conception shifted again to having more children. If the woman did not have a child, the pressure was to prove her fertility and provide lineage, however even where the couple had children they would be expected to have more. Ndoni (aged 25, one child) acknowledged reluctantly that if she married her new partner she would have to have another child with him. Khanyisile (aged 22, no child) who was married explained how this pressure continued if you were married without children. She was unable to conceive following an ectopic pregnancy, and reported feeling deeply saddened, as she wanted to be a mother. In addition she felt immense social pressure, judgement and blame, especially from the church who felt that as she was married she should have a baby. She was fortunate to be married to an older man in his 50’s who was supportive and did not add to this pressure and shame; he already had children from previous relationships, which may have led to him feeling more comfortable with not having more children.

### Pressure from partners

Some of the young unmarried women experienced pressure from their partners to conceive, however, many successfully resisted his wishes, usually surreptitiously. Yet, they all accepted that if he paid *lobolo*, or they married, they would have to have children with him. Five unmarried women specifically mentioned ignoring their partners’ pressure to conceive. The women’s ability to resist their partner’s pressure often increased as they grew older. Some women mentioned not being able to resist their partner’s pressure as teenagers, but that they could resist the pressure in their twenties, sometimes with the same partner. Their ability to resist partners was sometimes seen even within violent and controlling relationships. Strategies to avoid pregnancies varied, from delaying or avoiding sex, especially in teenage years, to secretly taking contraceptives.

Most women’s stories did not show that violent or controlling partners had more influence than non-violent partners over whether they conceived, and none of the women spoke about experiencing any violence related to their ignoring their partners’ wishes for children. Zoleka’s (aged 23, no child) partner was violent and controlling, and he wanted a child, she was ambivalent about motherhood, but despite him being violent, she felt no pressure to conceive because of his wishes. However, she did not use hormonal contraceptives as she believed the ancestors would decide if she conceived and she seldom used condoms. During the interview when asked about an area in her relationship where she felt she was able to make decisions Zoleka referred to the fact that despite her partner wanting a child, it had no influence over her:

Like this whole having a child business, I told him I will have a child when I am ready, I am not forced to have a child, if I fall pregnant then fine. But that doesn’t mean we must have sex everyday so that we can have a child.

Olwethu (aged 26) and Noluvuyo (aged 19), both with one child, were in violent relationships with partners who wanted more children, and both reported secretly using contraceptives to avoid conception. Noluvuyo had been with the same partner for 7 years, they had a child but did not live together, he wanted another child but she did not, he was physically and emotionally violent and on occasions threatened “to make her pregnant” and instructed her not to use contraceptives. The violence inflicted by their partners did not lead to these women acquiescing to their partner’s desire for more children, in both cases they continued using contraceptives secretly. While the men’s violence may have been a factor in discouraging the women from having more children with them, their violent acts and threats did not stop the women from preventing future pregnancies.

A range of factors contributed to women’s abilities to ignore their partner’s pressure for children, including age, already having a child and not cohabitating, in addition there were more important areas of conflict within their relationships. All the women who reported ignoring their partners’ pressure to conceive said that their relationships were quite turbulent, and that disagreements around conception and contraceptive use was a relatively insignificant area of conflict compared to other areas. Significant areas of conflict included infidelity by her partner and sometimes herself, his or her excessive drinking, him monitoring her cellphone usage and high levels of violence. These were the issues that the young women were concerned about and spoke about at length in interviews, photovoice sessions and during participant observations. In Sthelo’s (aged 26, no child) case her partner was regularly violent including an incident where he pulled a gun on her, and a second incident where they stabbed each other after she confronted him having found him in bed with someone else. This extreme violence did not make her fear his pressure for children, but rather appeared to make her even more dismissive of him, and his wishes for children.

The data suggests that women were more vulnerable to pressure from a partner in their teenage years. Five of the women reported pregnancies which were a result of partners’ pressure, four of which occurred when the women were teenagers, in most cases through him insisting on unprotected sex, when he knew she was not using contraceptives. Three of these resulted in miscarriages in their mid-late teens, Sthelo, Nomvelo and Zoleka. Of the other two, Noluvuyo, described how she fell pregnant at 17 when he wanted a child and she did not. She had managed to avoid pregnancy through delaying sexual debut for a long time, however shortly after agreeing to sex she conceived. Delaying sex to avoid conception was not sustainable within long-term relationships, where there was an expectation of sex. Langa (aged 23, one child) was older and did not yet have a child when she acquiesced to her partner’s pressure to conceive, she was ambivalent about conceiving and did not use contraceptives, during the photovoice baseline sessions she expressed this ambivalence “Sometimes I want a child, other times I don’t”. Her partner wanted a child, and refused to use condoms when she asked him to, this lead to a lot of tension. She said that eventually she relented to unprotected sex and became pregnant shortly thereafter, she was pregnant during her 12 month interview.

## Discussion

The young women’s experiences showed that while they were still teenagers and into their early twenties, and had not yet had a child, most of them were not particularly concerned about whether or not they conceived or became mothers. It was n not a major issue to them at this point of their lives, and they rarely thought about it. This is contrary to the discourse that assumes that young women want to prevent pregnancies and that it is their lack of agency that stops them from preventing these unwanted pregnancies [7, 8, 13]. This research concurs with other researchers who found that during women’s teenage years the normative pressure to conform and be like their peers, and prove their fertility resulted in many of the women conceiving young without giving it significant thought, and simply following the normative expectations [3, 5, 26].

As the women became older (into their mid-twenties), and/or after having a first child, some began to think about whether and when to have more children, and to reflect on how their lives might evolve over the next few years and how to accommodate babies within this. Among these women the normative pressures to conceive young had either been met, or if they had not as they grew older these pressures were significantly reduced. At this stage, the women began to talk about making choices and setting goals about whether or not to have more children, often deciding to delay further children until they had completed school, found good work or married. Their emerging actions to realise their goals usually involved avoiding sex or secretly using contraceptives, similar to what was seen in much literature [15]. In most cases the women did not discuss this with their partners, particularly in cases of violent partners, and they usually managed to ignore pressure from partners who wanted more children. This differed from some of the literature which found that pressure from partners to conceive had significant influence over young women [16, 26]. Nonetheless we saw that even where women said they wanted to delay further babies, they were often unable to act upon this. Even when resolute to prevent pregnancies, similar to other studies [3], in many cases the women found it challenging to consistently use contraceptives, and often decisions to avoid pregnancy were undermined by the challenges of regularly accessing and using contraceptives.

While theories of social norms and agency were both helpful in understanding young women’s reproductive decision-making, or lack of decision-making, we found the theories have different levels of relevance to the women, at different points in their lives. Social norm theory [21] is more relevant to explain the teenage women’s experiences, where the women felt normative pressures to have a baby while young. These young women followed what they believed others expected them to do, often giving very little thought to it, they believed their family and community expected them to be young mothers and so that is what they did. Whereas reproductive agency [9] became a more helpful framework later in their lives. Agency theory assumes women have a desire to set reproductive goals about motherhood, and related contraceptive use and fertility and recognises that they are often constrained from setting and achieving such goals. This became true for the women as they became older and began to want to plan whether and when to have babies, despite often facing challenges in acting upon these desires. This is similar to Stern et al’s (2016) finding that women sought less violent relationships and became more aware of their own sexual desires as they became older [38]. This is also consistent with the Theory of Planned Behavior (TPB) which notes that women’s behaviors are informed by their attitudes towards a behavior, subjective norms and perceived control over the behavior (agency) [24]. As women began to want to control their reproduction, some were still unable to do so as they did not perceive that they had meaningful control over it. As such, the young women’s inconsistent contraceptive use may be explained in part by the TPB, despite sometimes wanting to use contraceptives (individual attitude to the behavior), many women still did not feel they could effectively control their use of contraceptives due to lack of access, inadequate knowledge and insufficient power or agency within their relationships.

One interpretation of our findings was that women in their mid-20s were shifting towards behaviors more commonly associated with reproductive agency and Kabeer’s (1999) ideas of ‘identifying a goal and finding ways to act to achieve it’. However, even within the agency discourse their actions were more akin to Campbell and Mannell’s (2016) ‘distributed agency’ in that they were shifting towards new ideas around their reproduction and taking control to prevent pregnancies, however it was gradual and they used strategies that were meaningful and feasible within their context, such as secretly using contraceptives. Distributed agency recognises a wide range of actions that may be perceived as ‘smaller’ goals and actions, but which are realistic and achievable in, and shaped by, the women’s particular contexts. These acts are shaped by the women’s own experiences, for these women this included, their age, whether they already had children, their goals around completing their education, finding employment and their relationships. Distributed agency also recognises that agency is often fluid, women may have it at one point, and yet not at another. This was evident when the older women began expressing desires to control their fertility, in some cases they succeeded, and in others their fertility intentions were undermined by contextual factors such as the challenges of regularly returning for follow up contraceptive hormonal injections.

The study had some limitations, the fact that it was undertaken in only one area in South Africa, and the use of purposive sampling, means that it is not generalisable across the country, nonetheless the insights are important and may provide something to think about for those elsewhere. Further, as there were only two women who had not conceived we could not undertake a meaningful comparison between those who conceived versus those who did not. Nonetheless, the rich case studies enabled us to identify insights and understandings that support researchers to critically examine the experiences of young women in informal settlements. Additionally, the multiple interviews and forms of data collection enhances the validity of the findings.

## Conclusion

The dominant narrative around reproductive decision-making is focused on identifying ways to build women’s reproductive agency [7, 8, 13], however, these findings challenge us to reflect upon both social norm and agency theory. We found social norm theory fitted better for younger women (teenagers through to their early 20’s), while agency theory, particularly distributed agency was more meaningfully for older women (as they moved into their mid-twenties), although social norm theory still played a small role for some. The theory of planned behavior also proved helpful in understanding women’s beliefs in terms of their perception of where they had control, at times desiring to prevent pregnancies but not believing they could do this as they did not believe they could control their contraceptive usage. While we argue that these theories can be relevant at different stages of women’s lives we caution against an over-emphasis on one over the other, and also highlight that in many cases ideas of controlling one’s fertility and making active reproductive decisions were not common to young women’s lives, particularly so before having their first child, although this shifts for some women after having a child and as they grow older. In challenging contexts reproductive decision-making was a relatively small, and often unimportant, part of women’s daily decision-making.

This limited interest among many young many women to control their reproduction poses programmatic and policy challenges for health care activists, development workers, researchers and policy makers who aim to support women to think about, and act upon, their reproduction choices. Therefore we recommend those supporting young women to avoid early, unplanned motherhood, need to develop a more nuanced understanding of what is important to young women at different stages in their lives, in different contexts. Programmatic and research teams developing or implementing interventions need to start with formative research to ensure a thorough understanding of young women’s specific beliefs, experiences and priorities, in addition theories of change need to directly address these beliefs, experiences and priorities in order to be effective. Furthermore, policy and programmatic stakeholders need to ensure interventions and/or services are shaped to the specific interests and priorities of women at each stage of their lives; and must target social norms for young women who are not mothers and increasing agency for older women who already have children, currently many policies and programmes fail in this regard.

## Supporting information

S1 Appendix In-depth female baseline interview guide. (DOCX)

S2 Appendix In-depth females follow up 12 and 18m interview guide. (DOCX)

S3 Appendix Photovoice guide. (DOCX)

S1 Table One participants demographic characteristics. (DOCX)
