# Supplementary material for: Exploring young women’s reproductive decision-making, agency and social norms in South African informal settlements
Source: PLoS One. 2020 Apr 29;15(4):e0231181. doi: 10.1371/journal.pone.0231181 (PMC7190118; doi:10.1371/journal.pone.0231181)
Supplement: S1 Appendix — (DOCX) [file pone.0231181.s001.docx]

**Appendix One – SS/CF Women’s Qualitative Work**

**Guide for IDI at Baseline**

**First Interview**

***Overall question of interest:***

How does gender inequality, poverty and femininity shape women’s agency, and in turn how does women’s agency impact on their experiences of IPV and reproductive decision making?

This interview focuses on getting to know the young women:

**Question: Please tell us about yourself:**

Possible Probes:

- Where do you live
- Who do you live with
- What is your home like
- How long have you lived in this community? Where were you living before?
- Why did you move here?
- Do you enjoy living here? Why or why not

**Question: Please tell us about the people in your life:**

Note: for each relationship they discuss ask probes.

Possible Probes:

- Do you have a boyfriend(s)?
- If so please tell us about him? How old is he? Does he work?
- How often do you see him and what do you do when you see him?
- Do you enjoy spending time with him? Would you like to see him more often? If so what is stopping you from seeing him more?
- How did you meet?
- If you are living together how did that come about?
- Who else in your life helps you in times of crisis? Who can you turn to if you need to borrow R50?
- Are your parents still involved in your life? How?

**Question: Do you have children?**

Possible Probes:

- If so how many and ages? Do they have the same father?
- Are you still in a relationship with the father of the children?
- What role does the father play?
- Who helps you with you children? Who looks after them when you work?
- Are you breastfeeding? How do you feel about the idea of breastfeeding?
- Do you receive the child support grant?

**Questions: Please tell us about what you do on a daily basis**

Possible probes

- Briefly describe your average day
- What do you enjoy about it and what do you not enjoy

**Question:** **Tell me about what you do to get by/make money?**

(This may have been covered above)

Possible probes:

- For each ‘strategy’ ask about how successful it is
- For each ‘strategy’ ask about the difficulties of it
- Do you get money from people ever?
- What would be a ‘good’ job?
- Do you save money? How do you save and what are you saving for?

**Tell me about the food you eat, what do you eat, and why may you not eat for a day?**

Possible Probes:

- Do you generally get enough food to eat?
- Do you ever go hungry, why?
- If you go hungry, what do you try to do to get food? (Ask about how successful strategies are)

Who buys most of the food that you eat and where do they get it from?

**Question: Please tell us about the challenges in your life:**

Possible probes:

- Why is it such a challenge?
- What would make it possible for you to overcome this challenge?
- Can you do anything to change this challenge?

**Question: Tell me what kinds of life shocks you have experienced in the last two years, and what happened in your life as a result?**

Possible Probes:

- What happened – was it a death of someone important, a loss of a physical asset such as a house, an unexpected expense, an illness, a job loss in the family?
- For each shock, ask what did you do to cope – did you turn to someone for help? Find ‘emergency’ work? Borrow money?
- What happened after the shock was over – did you recover, or are there still problems?

**Question: why did you join the intervention and what do you hope might change following this intervention?**

Probes:

- Why did you join the intervention?
- What do you hope might change after the intervention? Please be specific.
- Where do you hope to be in 6 months’ time?

**Second Interview (approximately 1-2 weeks later)**

***Note: review transcript from first interview to ensure enough knowledge for a fruitful follow up interview***

***Overall question of interest:***

How does gender inequality, poverty and femininity shape women’s agency, and in turn how does women’s agency impact on their experiences of IPV and reproductive decision making?

This interview aims to get more in-depth.

**Question: Please tell us about what you have done that makes you really proud of yourself:**

Possible probes:

- What in particular made you so proud about doing it?
- How did you manage to do this thing?
- Are there people in your life that helped to make this possible?

The interview will then seek to explore widely held perceptions of femininity in the community and how the participant relates to this

**Question: What is it like to be a woman in this community?**

Possible probes:

- What do women do in this community?
- What are the most important characteristics of being a woman in this community?
- If someone does not conform to these characteristics of being a woman, are there problems?
- Does alcohol play a large role in the life of women in this community? Does it play a big role in your life?
- Does not having much money cause problems for you as a woman? How and why?

**Question: Are men violent in this community?**

Note: if the participant expresses perpetrating violence or experiencing violence explore this further.

Probes:

- Who are men violent towards?
- Why do you think they are violent to these people?
- Do men ever hit women? What does the community think?
- When are women violent? Who are they violent towards and why?

**Question: What type of man makes a good boyfriend?**

Note: if the participant talks about themselves/their boyfriend explore this

Probe:

- Should women receive money/food off their boyfriends?
- How do you think a good boyfriend should act/behave?
- Should girlfriends do cooking for their partners?
- Should a man know where his girlfriend is?
- Is it OK for a man have more than one girlfriend?
- Is it OK for a woman have more than one boyfriend?

The interview will then explore her intimate sexual relationships and reproductive decision making in detail

**Question: Tell me about the relationships you have with boyfriends/partners.**

Note: for each relationship they discuss ask probes.

Probes:

- Do you every give him money/food/gifts? Why?
- Do you every receive money/food/gifts? Why?
- How do you get along? What happens when you disagree?
- If he wants sex, but you do not, what happens?
- If you wanted to leave your boyfriend, what would happen?
- Have you ever been shouted at by a boyfriend? Has a boyfriend ever hit you? Why did it happen? How did you respond? What was the outcome?
- Has a boyfriend ever forced you to have sex when you did not want to? Please tell me what happened?

**Question: Tell me about your decisions around contraceptive use,**

Probes:

- Do you use condoms with your boyfriend? Why?
- What would happen if he asked to use a condom?
- What would happen if you asked to use a condom?
- Do you use any other form of contraceptive (e.g. injection, pill, inplant)? If so which one and for how long? If not, would you like to and what stops you from doing so? Tell me how you made the decision?

**Question: Tell me about your thoughts about whether to fall pregnant, and whether to keep a baby.**

Probes: (please recall from previous interview whether she has a child and ask appropriate questions)

- If you have a child, did you want to have the baby at the time of falling pregnant? Whose decision was it to keep the pregnancy? And who is the primary caregiver for your baby?
- If you do not have a child, would you like one now, and if so what is stopping you from having one?
- In your current relationship(s) do you ever talk about having a baby/child? Can you tell me about the discussion?
- If you want a child and your boyfriend does not, what do you think you will do?
- If your boyfriend wants a child and you do not, what do you think you will do?
- What things shape other young women’s decisions to have children in this community? Can you tell me a story about young women’s decisions about this?
- Do you and your friends discuss this much? If so, what do you talk about?

**Question: Tell me about your thoughts about abortions**

Probes:

- Have you ever had an abortion? If so whose decision was it, and how do you feel about the decision?
- Who do you think should make a decision about having an abortion?
- How do you feel about friends or other women who have abortions?

**Question: Let’s talk about HIV in your life. Have you had an HIV test, are you on HIV treatment and how do you feel about this?**

Probes:

- If you have had a test what made you decide to test?
- How did you respond when you got the result?
- If you are living with HIV how has this affected your life?
- If you are on HIV treatment how has this impacted on your life?
- If you are living with HIV and have a baby how has this impacted your decision about motherhood i.e.: whether to have children, whether to breastfeed etc

The interview will then explore her experiences of having power and control over her own life – whether she has agency

**Question: Tell me about your relationships and whether you have the power to make your own choices in your relationship(s)?**

Probes:

- In your current relationship(s) do you feel that you are able to make important decisions and have control? If so, please tell us what decisions these are.
- In your current relationship(s) do you feel that there are areas where you are NOT able to make important decisions and have control? If so, please tell us what decisions you do NOT have control over. For instance do you have control over decisions about how to spend your money, whether to go out in the evenings, how to raise your children etc
- Who is stopping you from making important decisions in your relationship? Why do you think s/he does this? And how does s/he stop you from making decisions?
- If you and your boyfriend(s) disagree on an important issue what happens? What happens when you argue? How is it resolved? How would you like it to be sorted out?
- Do you want to make more decisions in your relationship then you currently do? If so what decisions would you like to make?
- Are there any other things beyond the person mentioned above that stops you from being in control in your relationship?
- What would you do differently if you were able to make all the decisions you want to in your relationship? How might your life be different?
- Do you and your friends discuss this? If so what do you talk about?

**Question: How do you decide whether to stay in a relationship, to leave it or to start a new relationship?**

Probes:

- How did your current relationship(s) begin? Would you say you had control over whether to begin the relationship i.e. did you choose this relationship?
- Why do you stay in the relationship?
- If you wanted to leave the relationship would you be able to do so? If not what would stop you?
- Have you ever ended a relationship? What made you end it, and did you feel it was your decision? How did you end it? How did you feel about ending it?
- Have you ever stayed in a relationship that you wanted to leave? If so what made you stay?
- If you met someone that you wanted a relationship with would you be able to tell him, and to start a new relationship?
- How would you start a new relationship?
- What might stop you from starting a new relationship?
