# Supplementary material for: Exploring young women’s reproductive decision-making, agency and social norms in South African informal settlements
Source: PLoS One. 2020 Apr 29;15(4):e0231181. doi: 10.1371/journal.pone.0231181 (PMC7190118; doi:10.1371/journal.pone.0231181)
Supplement: S2 Appendix — (DOCX) [file pone.0231181.s002.docx]

**Appendix Two – SS/CF Women’s Qualitative Work**

**Guide for IDI at 12 and18 months**

***Note: this interview will build upon the knowledge we have been gathering over the 18 month period in the participant observations and photo voice process.***

***Overall question of interest:***

What has changed, or not, as a result of the intervention and why?

The interview will begin with a discussion of the intervention, how they experienced it, and what they are now using it for.

**Question: Let’s talk about your experience of the intervention. How did you find the intervention? Why?**

Probes:

- What was your experience of the workshops? Which sessions did you attend? Which do you remember? Which appealed to you the most and why?
- Were you able to participate in discussions? How did it feel? What were the difficulties? Did it change with time?
- Which sessions were difficult in terms of your own feelings or in terms of the group not wanting to discuss sensitive issues? Did discussion of difficult things get easier over time? Were you able to discuss personal issues/stories? Were other participants able to discuss personal issues?
- Is there anything about the intervention you would change?
- Would you recommend that your friends should have also participated in the intervention?

The interview will now look at the impact of the intervention on how people get by/survive.

**Question:** **Tell me about what you do to get by/make money?**

Possible probes:

- For each ‘strategy’ ask about how successful it is
- For each ‘strategy’ ask about the difficulties of it
- Do you save money? How do you save and what are you saving for?

Probe for any changes:

- How has this changed since the intervention?
- What has been hard about trying to change your livelihood?
- How did the intervention assist you in these new activities?

**Question: What are the important relationships with friends, families or NGOs, you have that help you get by/make money?**

Possible probes:

- Who can you borrow money off if you need R50?
- In what ways have any of these relationships helped you in the last few months? And how and why did the help come about (did you initiate it?) and what happened?

Probe for any changes:

- How has this changed since the intervention?
- Do you have more support in difficult times? If so why?
- How did the intervention assist you in these new activities?

**Tell me about the food you eat, and why may you not eat for a day?**

Possible Probes:

- Do you generally get enough food to eat?
- Do you ever go hungry, why?

Probe for any changes:

- How has this changed since the intervention? Are you eating better and going hungry less often? If so what is different?
- How did the intervention assist you in coping differently?

The interview will briefly discuss the participants everyday life – to ‘paint the picture’ of her life, and how it has changed following the intervention

**Questions: Please tell us about your life, what you do on a daily basis and how your life has changed following the intervention?**

Probes:

- Are you in the same relationship you were in when the intervention began? If not how did it end? Whose decision was it to end it? Are you happy with the decision?
- If you have children, has the fathers involvement changed since the intervention, and if so why? Who else helps you to look after the child?
- What has changed in your life following the intervention 18 months ago?
- What do you still hope will change in your life? Please be specific.

The interview will then seek to explore widely held perceptions of femininity in the community and how the participant relates to this

**Question: What is it like to be a woman in this community?**

Possible probes:

- Do you think you have become a different kind of women since the intervention? If so please describe in what ways.
- Has your alcohol use changed since the intervention, how? And how has this impacted on your life?
- Should women have many partners, why? Has your opinion on this changed since the intervention? If so why?

**Question: What type of man makes a good boyfriend?**

Note: if the participant talks about themselves/their boyfriend explore this

Probe:

- How do you think a good boyfriend should act/behave? How has your view on this shifted since the intervention?
- Should girlfriends do cooking for their partners? How has your view on this shifted since the intervention?
- Should a man know where his girlfriend is? How has your view on this shifted since the intervention?

The interview will then explore her intimate sexual relationships and reproductive decision making in detail

**Question: Tell me about the relationships you have with boyfriends/partners.**

Note: for each relationship they discuss ask probes.

Probes:

- How often do you see them?
- Do you ever give him money/food/gifts? Why?
- Do you ever receive money/food/gifts? Why?
- How do you get along? Are there any issues you disagree on or not? What happens when you disagree? Has this changed since the intervention? Do you disagree less? Do you handle disagreements differently? If so how?
- If he wants sex, but you do not, what happens? Has this changed since the intervention? Do you handle this differently? If so how?
- If you are not at home or don’t answer the phone when he calls, what happens?
- Since the intervention has a boyfriend hit you? Why did it happen? How did you respond? What was the outcome?
- Do you spend more time with your main partner since the intervention? Why?
- Do you think you have more or less casual partners since the intervention? Why?
- Since the intervention has a boyfriend forced you to have sex when you did not want to? Please tell me what happened?

**Question: Tell me about your decisions around contraceptive use,**

Probes:

- Do you use condoms with your boyfriend? Why?
- What would happen if he asked to use a condom? What would happen if you asked to use a condom? How has this changed following the intervention?
- Do you use any other form of contraceptive (e.g. injection, pill, inplant)? If so which one and for how long? If not, would you like to and what stops you from doing so? Tell me how you made the decision? How has this changed following the intervention?

**Question: Tell me about your thoughts about whether to fall pregnant, and whether to keep a baby.**

(If you know this from ongoing observations do not ask)

Probes:

- Have you fallen pregnant since the intervention? If so did you keep the pregnancy and have a live birth? How did you make this decision? Was it your decision or someone else’s? Did the intervention influence your decision in any way? If so how?
- And who is the primary caregiver for your baby?
- If you do not have a child, would you like one now, and if so what is stopping you from having one?
- In your current relationship(s) do you ever talk about having a baby/child? Can you tell me about the discussion? And has this discussion shifted following the intervention?

**Question: Tell me about your thoughts about abortions**

(If you know this from ongoing observations do not ask)

Probes:

- Have you had an abortion since the intervention? If so whose decision was it, and how do you feel about the decision?
- Who do you think should make a decision about having an abortion?
- How do you feel about friends or other women who have abortions?

**Question: Let’s talk about HIV in your life. Have you had an HIV test in the last 18 months, and how do you feel about this?**

Probes:

- If you have had a test in the last 18 months what made you decide to test?
- How did you respond when you got the result?
- How has this impacted on your life? Are there any ways in which you may be coping better as a result of the intervention, please provide examples.

The interview will then explore her experiences of having control over her own life – whether she has agency

**Question: Tell me about your relationships and whether you have the power to make your own choices in your relationship(s)? And how this has shifted since the intervention.**

Probes:

- In your current relationship(s) do you feel that you are able to make important decisions and have control? If so, please tell us what decisions these are. Has this shifted since the intervention, and in what way?
- In your current relationship(s) do you feel that there are areas where you are NOT able to make important decisions and have control? If so, please tell us what decisions you do NOT have control over. How has this shifted since the intervention, and in what way?
- Who is stopping you from making important decisions in your relationship? How has this shifted since the intervention, and in what way? And how do you feel about this change?
- If you and your boyfriend(s) disagree on an important issue what happens? What happens when you argue? How is it resolved? How would you like it to be sorted out? How has this shifted since the intervention, and in what way? And how do you feel about this change?
- Do you want to make more decisions in your relationship then you currently do? If so what decisions would you like to make? How has this shifted since the intervention, and in what way? And how do you feel about this change?
- Where you able to change what you did with existing boyfriend(s) or only with new ones? Please explain why.
- What would you do differently if you were able to make all the decisions you want to in your relationship? How might your life be different?

**Question: How do you decide whether to stay in a relationship, to leave it or to start a new relationship?**

Probes:

- If you are in a new relationship since the intervention, please tell us how it began? Would you say you had control over whether to begin the relationship i.e. did you choose this relationship?
- Do you feel this relationship is different from previous ones? Do you feel you can make more decisions and have more power?
- Are you in the same relationship that you were in during the intervention 18 months ago? If not who ended it and how did you feel? Why did it end?
- If you wanted to leave the relationship would you be able to do so? If not what would stop you? How has this shifted since the intervention, and in what way?
- Have you ever ended a relationship since the intervention? How did you end it? What role do you think your participation in the intervention played in your decision to end it?
- Have you stayed in relationships that you would like to leave? If so what made you stay?
- If you met someone that you wanted a relationship with would you be able to tell him, and to start a new relationship? How has this shifted since the intervention, and in what way?
- How would you start a new relationship?
- What might stop you from starting a new relationship?

The interview will directly explore what helped and hindered her change following the intervention.

**Question: Do you think you are able to make more decisions in your intimate relationships? If so, what do you think made it possible for you to change?**

- What was it about the intervention that helped you to have more power in your relationship?
- How did their partner, family and/or community react when you began to make important decisions about your relationship?
- At what point did you put things you learned from the intervention into practice? For example, while you were still attending the workshops, or after the workshops had finished?
- Have you talked about this with any of your friends? What do they think about your change?
- Now that you are doing something differently what has that change meant for you? What else has changed in your life following that change?

**Question: In your intimate relationship when you have wanted to assume equal power what challenges have you experienced?**

- For each challenge ask them:
  - Why have things not changed?
  - Do you want to change things in the future?
  - What do you need to support you to change?
  - What would be different for if that change occurred?

**Question: What would you like to be doing in the next six months?**
