# Supplementary material for: Exploring young women’s reproductive decision-making, agency and social norms in South African informal settlements
Source: PLoS One. 2020 Apr 29;15(4):e0231181. doi: 10.1371/journal.pone.0231181 (PMC7190118; doi:10.1371/journal.pone.0231181)
Supplement: S3 Appendix — (DOCX) [file pone.0231181.s003.docx]

**Appendix Three – SS/CF Women’s Qualitative Work**

**Guide for Participatory research methods**

**Photovoice**

**Objective:** To explore young women’s reflections and experiences of reproductive decision-making with a small group of women using the photovoice technique, which is a participatory technique based on critical consciousness where women are supported to use photographs to represent their lived experience and aspirations.

This work will supplement the in-depth interviews and participant observations, providing an opportunity to record and analyse the experiences of the young women.

We will undertake this work with 8 women who participated in the IDI’s, and who are not involved in the participant observation process.

**Frequency**:

The participants that they will be asked to be involved in this process twice; at baseline, and 18 months, each time period involves two activities/sessions. The baseline process is split into two separate sessions, about two weeks apart.

**Language:**

All discussions will take place in isiZulu, however the facilitators will be bilingual and as such this guide does not need to be translated.

**Cameras:**

We will supply small disposable cameras, however, some people may choose to use their own cellphone cameras (where they have them).

**Ethics:**

Remember at the start of each session to reinforce that people do not have to participate if they do not want to. This will not affect their participation in the wider trial. They must sign the informed consent form.

**Materials needed:**

- Laptop
- Portable printer, ink, paper
- Recording equipment
- One facilitator

**Baseline Work – focus on the notion of power in their lives**

**Session one – Training the participants and critical discussion**

**Purpose:**

- To generate discussion about power, and where she may have some power in her relationship, in order to stimulate ideas and reflections; and
- Train the participants on how to think about the composition and message of their photos, how to take the photos and ethics of taking photos

**Time needed:** This process will take 3 hours.

**Key Steps**:

Introduce the whole process and clearly outline the purpose of this session, and the “homework”.

**Facilitate a participatory group discussion where you support the group to reflect on the following ideas**: (1.5 hour)

- What does power mean to you?
- How do you think the following ‘types’ of power might be different – ‘power over’, ‘power to’ and ‘power with’?
- Can you think of any situations where you might have experienced any of these types of power? Please explain.
- In your current relationship(s) do you feel that you have the ‘power to’ make important decisions? If so, please tell us what decisions these are. Please push the reflections around:
  - whether they have the power to make decisions about whether and which contraceptives to use and when,

whether and when to conceive,

whether to terminate a pregnancy, and

whether to keep a baby?

Wrap the discussion up by reminding participants that photovoice is a powerful tool to explore all these ideas, and that they will be using photovoice to capture their experiences and ideas around these issues.

**Run a discussion introducing the idea of photovoice and reflecting on how a photo can ‘tell a story’, and the importance of each participant deciding what story they want to tell** (1 hour)

Show the participants 3-5 photos and ask them what they think is being relayed by the image- lead a discussion on how powerful images can be.

Lead a reflection about how photos can both reflect a situation as it is and also show aspirations and hopes and dreams.

Explain to participants that we are using this process to do two things:

to reflect on their own experiences around having ‘power to’ make critical decisions in their relationships and

in the next session to reflect on where they feel their boyfriend has ‘power over’ their critical decisions and also their hopes and aspirations in terms of where they would like to have ‘power to’ make decisions in their relationships and ‘power with’ their boyfriend.

Teach the women how to use the cameras (where necessary)

Explain that they can take as many photos as they like, however we would only like them to bring between 4-7 to work with in the next session.

**Outline the steps from here**: (30 minutes)

Remind participants that we want their photos to reflect on the earlier discussion.

Ask them to think about the photo story she wants to tell, what story does she want to share. We would like her to tell the photo story (using photos) of:

What she thinks power is

Instances where she has the ‘power to’ make important decisions in her relationship: this could be about anything related to the topic e.g. your own contraceptive use, falling pregnant, terminating, giving birth, etc?

Spend one day reflecting on your message/story and then decide where you might be able to take these images.

Allow yourself a few days to take the photos (allow extra days in case of poor weather, illness, emergencies etc)

Come back in two weeks with the images on your camera.

**Session two – Designing your photo story:**

**Purpose:**

- To share the photos that most powerfully communicate participant’s messages/story about reproductive decision making; and
- Document a narrative to support the photo story.

**Time needed:** This process will take 2.5 hours, and will be one-two weeks after the first session.

**Key Steps**:

Remind the participants of the discussion 1-2 weeks earlier – remind them of the issues they were discussing and the focus and purpose of the photovoice exercise. This ‘refresher’ should be led by the participants and not a didactic summary by the facilitator.

- Each participant will come with the 3-5 photos that tell her story. They will be printed immediately.
- Give participants 30 minutes to develop photo-posters, they should arrange their photos and write short descriptions (in isiZulu or English).
- Each participant then shows their photo-poster and describes it, followed by group discussions and reflections.
- The session will be recorded to capture the story. It will be transcribed and translated into English.

**Facilitate a brief participatory group discussion where you support the group to reflect on the following ideas**: (30 mins)

- Reflect briefly again on: what power mean to them, and how the ‘types’ of power might be different – ‘power over’, ‘power to’ and ‘power with’? [They may already be thinking about this differently]
- In your current relationship(s) do you feel that there are areas where your boyfriend has ‘power over’ your decisions and you are NOT able to make important decisions? If so, please tell us what decisions you do NOT have power over. Please push the reflections around:
  - do you have control over decisions about how to spend your money,
  - whether to go out in the evenings
  - whether they have the power to make decisions about whether and which contraceptives to use and when,

whether and when to conceive,

whether to terminate a pregnancy, and

- - whether to keep a baby?
- Do any other people in your life have ‘power over’ important decisions in your life? Who are they and what do they have ‘power over’?

If someone else makes these decisions for you do you resit it at all, and if so how?

In an ideal world which of these decisions would you like to be making?

What decisions in your relationship would you like to have the ‘power to’ make?

What decisions would you like to make with your boyfriend?

What would need to change to enable you to make those decisions?

Wrap the discussion up by reminding participants that photovoice is a powerful tool to explore all these ideas, and that they will be using photovoice to capture their experiences and ideas around these issues.

**Brief the women to take photos to represent this.**

**Session three – Designing your second photo story:**

**Purpose:**

- To follow up on the discussion about power, and where she does not have power in her relationship, in order to stimulate ideas and reflections;
- Her aspirations around what it would be like to have ‘power to’ make critical decisions in her relationship; and
- Design second photo poster, with a brief narrative reflecting on a ‘lack of power’.

**Time needed:** This process will take 3 hours, and will be one-two weeks after the second session.

**Key Steps**:

Remind the participants of the discussion 2 weeks earlier – remind them of the issues they were discussing and the focus and purpose of the photovoice exercise. This ‘refresher’ should be led by the participants and not a didactic summary by the facilitator.

- Each participant will come with the 3-5 photos that tell her story. They will be printed out.
- Give participants 30 minutes to develop photo-posters, they should arrange their photos and write short descriptions (in isiZulu or English).
- Each participant then shows their photo-poster and describes it, followed by group discussions and reflections.
- The session will be recorded to capture the story. It will be transcribed and translated into English.

**Eighteen month follow up**

The same 8 women will be asked to be involved in another photovoice project 18 months after the intervention. The same approach will be used (with refresher camera training).

The discussions will focus on the same topics but will shift to exploring what has changed, and not, 18 months later, and what enabled or hindered the changes. They will be asked to tell their story again, to reflect any changes following the intervention.

We will wrap this process up in the final workshop by bringing all three photovoice stories and allowing extra time – we will facilitate a discussion where the women themselves reflect on any changes they see in these photovoices over 18 months.
