# Supplementary material for: Exploring young women’s reproductive decision-making, agency and social norms in South African informal settlements
Source: PLoS One. 2020 Apr 29;15(4):e0231181. doi: 10.1371/journal.pone.0231181 (PMC7190118; doi:10.1371/journal.pone.0231181)
Supplement: S1 Table — (DOCX) [file pone.0231181.s004.docx]

**S4 Table one: Participants demographic characteristics (all names are pseudonyms)**

| **Name (age range at baseline)** | **Ever pregnant,** | **Children?** | **Intimate relationship at baseline?** | **Lifetime experience of IPV?** |
| --- | --- | --- | --- | --- |
| Enhle  ( 20-25) | Yes | One | No | Physical IPV |
| Olwethu (26-29) | Yes | One | Yes | Physical and emotional IPV |
| Ntombi (20-25) | Yes | One | No | Physical and emotional IPV |
| Nkanyezi (unknown) | Yes | Three | No | No |
| Thembeka (26-29) | Yes | Three ( | Yes | Emotional IPV |
| Thobile (20-25) | Never | None | Recently ended | Sexual and emotional IPV |
| Sebenzile (26-29) | Yes | One | Yes | Emotional IPV |
| Sthelo (26-29) | Yes, miscarried | None | Yes | Physical and emotional IPV |
| Noluvuyo (18-19) | Yes | One | Yes | Physical and emotional IPV |
| Thuleka (18-19) | Never | None | Yes | Physical IPV |
| Ndoni  (20-25) | Yes | Two oldest died during research period | Yes | Physical and emotional IPV |
| Langa  (20-25) | Yes, miscarried . Pregnant during research period | One | Yes | Physical IPV |
| Khanyisile (20-25) | Yes, ectopic pregnancy that miscarried | None | Yes | No IPV, physical and sexual non-partner violence |
| Nomvelo (20-25) | Yes, miscarried Pregnant during research period | One | Yes | No |
| Zoleka (20-25) | Yes, miscarried | None | Yes | Physical and emotional IPV |
